# Water deficit mechanisms in perennial shrubs *Cerasus humilis* leaves revealed by physiological and proteomic analyses

**DOI:** 10.1186/s12953-017-0117-1

**Published:** 2017-05-08

**Authors:** Zepeng Yin, Jing Ren, Lijuan Zhou, Lina Sun, Jiewan Wang, Yulong Liu, Xingshun Song

**Affiliations:** 1 Department of Genetics, College of Life Science, Northeast Forestry University, Harbin, 150040 People’s Republic of China; 2 State Key Laboratory of Tree Genetic sand Breeding, Northeast Forestry University, Harbin, 150040 People’s Republic of China; 3 Horticulture Department, College of Horticulture, Shenyang Agricultural University, No. 120 Dongling Road, Shenhe District, Shenyang, 110866 People’s Republic of China; 4 College of Food Science; Key Laboratory of Dairy Science, Ministry of Education, Synergetic Innovation Center of Food Safety and Nutrition, Northeast Agricultural University, Harbin, Heilongjiang 150030 People’s Republic of China; 5 Forest Engineering and Environment Research Institute of Heilongjiang Province, No. 134 Haping Road, Nangang District, Harbin, Heilongjiang 150081 People’s Republic of China

**Keywords:** *Cerasus humilis*, Proteomics, ROS, Water deficit, Perennial shrubs, qRT-PCR

## Abstract

**Background:**

Drought (Water deficit, WD) poses a serious threat to extensively economic losses of trees throughout the world. Chinese dwarf cherry (*Cerasus humilis*) is a good perennial plant for studying the physiological and sophisticated molecular network under WD. The aim of this study is to identify the effect of WD on *C. humilis* through physiological and global proteomics analysis and improve understanding of the WD resistance of plants.

**Methods:**

Currently, physiological parameters were applied to investigate *C. humilis* response to WD. Moreover, we used two-dimensional gel electrophoresis (2DE) to identify differentially expressed proteins in *C. humilis* leaves subjected to WD (24 d). Furthermore, we also examined the correlation between protein and transcript levels.

**Results:**

Several physiological parameters, including relative water content and Pn were reduced by WD. In addition, the malondialdehyde (MDA), relative electrolyte leakage (REL), total soluble sugar, and proline were increased in WD-treated *C. humilis*. Comparative proteomic analysis revealed 46 protein spots (representing 43 unique proteins) differentially expressed in *C. humilis* leaves under WD. These proteins were mainly involved in photosynthesis, ROS scavenging, carbohydrate metabolism, transcription, protein synthesis, protein processing, and nitrogen and amino acid metabolisms, respectively.

**Conclusions:**

WD promoted the CO_2_ assimilation by increase light reaction and Calvin cycle, leading to the reprogramming of carbon metabolism. Moreover, the accumulation of osmolytes (i.e., proline and total soluble sugar) and enhancement of ascorbate-glutathione cycle and glutathione peroxidase/glutathione s-transferase pathway in leaves could minimize oxidative damage of membrane and other molecules under WD. Importantly, the regulation role of carbohydrate metabolisms (e. g. glycolysis, pentose phosphate pathways, and TCA) was enhanced. These findings provide key candidate proteins for genetic improvement of perennial plants metabolism under WD.

**Electronic supplementary material:**

The online version of this article (doi:10.1186/s12953-017-0117-1) contains supplementary material, which is available to authorized users.

## Background

Among potential abiotic stresses, drought (water deficit, WD) is considered to have the largest effect on agricultural productivity, which limits plant growth, distribution and crop yield worldwide [1, 2]. It is estimated that the droughty terrestrial areas will redouble by the end of the 21st century [3]. Thus, it is extremely urgent to determine the mechanisms of plant respond to drought and improve the drought tolerance ability.

During drought stress, a series of metabolic alterations occur, including overproduction of reactive oxygen species (ROS), photoinhibition, denaturation of some proteins such as chloroplast proteins, damage to biofilm structure and functions, and inhibition in protein synthesis [1, 4, 5]. Plants employed series of strategies in response to WD, such as morphology, and physiology metabolisms. The imbalanced light energy conversion and carbon fixation in photosynthetic system may cause accumulation of ROS in plant cells, leading to photo-oxidation damages [2]. An excess of ROS production can lead to oxidative stress in plants and negatively impact the normal function of cells [4], which can damage DNA, lipids and proteins [6, 7]. ROS scavenging ability and subsequent injury-reducing effects may correlate with the tolerance to WD [8]. Both enzymatic and non-enzymatic defense systems have evolved in plants for scavenging and detoxifying ROS. The main non-enzymatic antioxidants in plants are soluble ascorbate and glutathione [9]. ROS scavenging enzymes such as ascorbate peroxidase (APX), superoxide dismutase (SOD), catalase (CAT) and peroxidase (POD) also play a very important role. In addition, the accumulation of various osmolytes, such as proline and soluble sugar, play an important protective role during WD, which result in the decrease of osmotic potential [10].

Although there are some researches in woody plants responses to drought in morphological and physiological level [11, 12], few studies were reported on molecular metabolisms. It has been shown that the genes/proteins were induced by WD either directly connected to stress response or implicated in the regulation of gene expression and signal transduction [13–15]. These studies provided important information for understanding WD-responsive gene functions. However, changes at the mRNA and metabolite levels do not always reflect changes at the protein level [16, 17]. Thus, studying the protein level changes in response to WD is important in woods. Proteomics technologies allow a high-throughput and systemic overview of the cellular physiology in a holistic manner to underscore the underlying metabolic and regulatory mechanisms [18]. It has been reported that WD altered the abundance of proteins involved in carbohydrate and energy metabolism, cellular detoxification, protein processing and degradation, signal transduction, and cell wall strengthening. Most of the previous work on WD related proteomics was performed on annual crops [19]. However, very limited proteomic information on perennial shrubs responses to WD is available. Perennial plants may express stress-responsive proteins associated with long-term adaptation or stress survival, as they must endure/persist through the stress period, unlike annual crops which produce seeds and may die in the case of severe WD [20, 21]. Thus, developing an adaptation mechanism is critical for the survival of perennial plants in WD environments.

Chinese dwarf cherry (*Cerasus humilis* (Bge.) Sok.), a species of perennial shrubs, originates in the north of China [22–24]. As most perennial dwarf shrubs in the world, *C. humilis* have the characteristics of being drought-, saline-, alkali-, cold- and sterile resistant, all of which endow the species considerable adaptabilities [23, 24]. Our previous studies showed that exogenous small aliphatic amines (spermidine and spermine) and microorganisms (e.g. photosynthetic bacteria) can alleviate the WD-induced oxidative stress in *C. humilis* [24]*.* Besides, the increased expression of *vde*, a gene encoding violaxanthin de-epoxidase in xanthophyll-cycle, confers a great capacity of photoprotection and thus contributes to the survival *C. humilis* against WD [23]. Although some physiological data and certain genes already give us some information, comparative proteomic analyses can deepen our understanding of plant stress acclimation/tolerance acquisition by providing a detailed picture of functional proteins in *C. humilis* under WD conditions.

Our objectives were to discover the WD-responsive characteristics of *C. humilis* by using combined physiological and comparative proteomic approaches. We also aimed to determine the role of ROS during this process. To address these questions, we (1) evaluated changes in gas exchange, the enzymes of antioxidants related to ROS, and the osmolytes protections, (2) carried out 2-DE based proteomics analyses the different proteins at different time point after WD, (3) investigated the expression changes of some raleted genes by qRT-PCR. These study lead to a better understanding of molecular mechanisms in perennial shrubs under WD.

## Methods

### Plant material and treatments

Cuttings of *C. humilis* obtained from HuaiRou district, Hebei province, China, were used in the present study. Each cutting was transplanted into the container (35 × 35 × 25 cm) filled with organic soil, irrigated regularly by Hoagland solution under a 12 h photoperiod at temperatures ranging from about 17–25 °C, photosynthetic photon flux density (PPFD) of 600 μmol m^-2^ s^-1^ and the relative humidity of 70–75% in the greenhouse. Seedlings at the 25–35 leaf stage were divided into two groups: well-watered plants were irrigated by water every two days (control), and water-deficit (WD) plants did not receive water. All measurements of physiological parameters were carried out on the youngest fully expanded leaves, with at least ten plants per-treatment.

### Determination of Relative water content (RWC), MDA, and relative electrolyte leakage (REL)

RWC was calculated as follows: RWC = [(FW - DW)/(SW - DW)] × 100. Fresh weight (FW) was measured immediately after harvesting, and saturate weight (SW) was measured right after saturation state immersed in distilled water. Then, the samples were oven-dried at 80 °C for 15 min, then vacuum-dried at 60 °C to constant weight and the DWs were recorded. The REL was measured by an electrical conductivity method [25].

### Measurement of photosynthesis and chlorophyll fluorescence

Net photosynthetic rates (Pn), stomata conductance (Gs), and intercellular CO_2_ (Ci) of leaves were determined during 8:30–11:30 h using a gas-exchange system (LI-6400; LICOR Biosciences, Lincoln, USA) on 24 days WD treatment. The photosynthetically active radiation was 1000 μmoL m^-2^ s^-1^ (saturation light). The ambient CO_2_ concentration was 360 ± 10 μmol moL^-1^, and the air temperature and humidity were about 24 °C and 50%. Measurements were repeated at least five times for each treatment and the averages were recorded.

The maximum photochemical efficiency of PSII (Fv/Fm) was measured using a pulse modulation chlorophyll fluorometer (FMS-2, Hansatech, UK) after 30 min dark adaptation [26].

### Determination of total soluble sugar, proline, H_2_O_2_ content and O_2_^-^ generation rate

Total soluble sugar and proline contents were determined using an anthrone reagent and ninhydrin reaction, respectively, as previously described [27].

To evaluate the levels of ROS in leaves, H_2_O_2_ content and O_2_^-^ generation rates were measured. Briefly, leaf tissue was ground in 0.1% trichloroacetic acid. The homogenate was centrifuged at 15,000 g for 15 min at 4 °C and the supernatant was collected for H_2_O_2_ measurement. H_2_O_2_ content was determined spectrophotometrically after reaction with potassium iodide [28], and O_2_^-^ generation rates were measured using a hydroxylamine oxidization method [29].

### Determination of antioxidant enzyme activities

The activity of antioxidant enzyme assays, including superoxide (SOD), catalase (CAT), peroxidase (POD), ascorbate peroxidase (APX), monodehydroascorbate reductase (MDHAR), dehydroascorbate reductase (DHAR), glutathione reductase (GR), glutathione S-transferase (GST), glycolate oxidase (GO) and glutathione peroxidase (GPX), was assayed following the method of [30] and [24]. In all enzyme preparations, protein was quantified following by [31] using bovine serum albumin as a standard.

### Protein sample preparation, 2DE, and image analysis

Protein extraction and two-dimensional electrophoresis (2DE) separation were performed as previously described, with minor modifications [30]. Briefly, treated leaves (1g) was ground in liquid nitrogen, and total soluble proteins were extracted at 4°C in 8 mL of extraction buffer containing 100 mM Tris-HCl buffer (pH 8.8),10 mM EDTA, 0.9 M sucrose, and 0.4% mercaptoethanol. Homogenates were centrifuged at 15 000g for 15 min at 4°C, and the supernatants were added to 5 vol of 100 mM ammonium sulfate/methanol. Samples were maintained at −20 °C for 4 h and then were centrifuged at 20 000 g for 15 min at 4 °C. The resulting pellets were washed with 80% acetone containing at −20 °C for 1 h, and the 100% acetone wash once after centrifugation. The final pellets were vacuum-dried and dissolved in 7 M urea, 40 mM DTT, 4% (w/v) CHAPS, and 2% (w/v) ampholyte (pH 3 − 10). Samples in ampholyte were vortexed thoroughly for 1 h at room temperature and then were centrifuged at 35 000 g for 20 min at 20 °C. Supernatants then were collected for 2DE experiments [32]. Protein concentration was determined using a Quant-kit according to manufacturer’s instructions (GE Healthcare, USA). Extracted proteins were first separated by isoelectric focusing (IEF) using gel strips (pH 4-7 linear, 13 cm) (GE Healthcare,USA). Following IEF, proteins were separated by sodium dodecyl sulfate-polyacrylamide gel electrophoresis (SDS-PAGE) using 12.5% (w/v) polyacrylamide. Gel strips then were rehydrated in 450 μL of dehydration buffer containing 1600 μg of total proteins and a trace of bromophenol blue for 26 h. Gel strips were focused at 80 kV/h and 20 °C using the PROTEAN IEF system (Bio-Rad,USA) and then were equilibrated for 15 min in equilibration buffer (6 M urea, 0.5 M Tris [pH 8.8], 2% [w/v] SDS, 30% [v/v] glycerol). Gel strips then were placed over 12.5% (w/v) SDS-PAGE gels for 2DE. Gel electrophoresis was performed at 25 mA for 5 h. Gels were stained using Coomassie Brilliant Blue (CBB). After staining, gels were scanned using an ImageScanner III (GE Healthcare, USA) at a resolution of 300 dpi and 16-bit grayscale pixel depth. The images were analyzed with ImageMaster 2D software (version 6.0) (GE Healthcare, USA). The average vol% values were calculated from three technical replicates to represent the final vol% values of each biological replicate. The volume of each spot changed more than 1.5-fold among the treatments and a *p* < 0.05 were considered to be differentially expressed spots.

### Protein identification and database searching

Protein spots displaying significant changes in abundance were excised manually from colloidal CBB stained 2DE gels using sterile pipette tips. Briefly, spots cut out of the gels were destained twice with 100 mM NH_4_HCO_3_, 50% ACN at 37 °C for 20 min in each treatment. After dehydration with 100% ACN and drying, the gel pieces were pre-incubated in 10-20 μL of trypsin solution (10 ng /μL) for 1h. Then adequate digestion buffer (40 mM NH_4_HCO_3_, 10% ACN) was added to cover the gel pieces, which were extracted using Milli-Q water followed by double 1h extraction with 50% ACN and 5% TFA. The combined extracts were dried in SpeedVac concentrator (Thermo Scientific) at 4 °C. The samples were then subjected to mass spectrometry [30].

The MS spectra were acquired using a MALDI-TOF-MS/MS (AB SCIEX TOF/TOF: trademark: 5800 system). A Mass standard kit (Applied Biosystems, USA) and a standard BSA digest (Sigma-Aldrich, USA) were used for MS and MS/MS calibrations and fine-tuning the resolution and sensitivity of the system as previously described [33]. The mass error was below 30 ppm at both MS and MS/MS mode and the resolution was more than 25 000. The MS/MS spectra were searched against the NCBInr protein databases (http://www.ncbi.nlm.nih.gov/)(5,222,402 sequence entries in NCBI) using Mascot software (Matrix Sciences, London, UK). The taxonomic category was green plants. The searching criteria were according to [34]. The searching criteria include mass tolerance for precursor ions of 0.3 Da, mass tolerance for fragment ions of 100 ppm, one missed cleavage allowed, carbamidomethylation of cysteine as a fixed modification, and oxidation of methionine as a variable modification. To obtain high confident identification, proteins had to meet the following criteria: the top hits on the database searching report, a probability-based MOWSE score greater than 43 (*p*-value < 0.01), and more than two peptides matched with nearly complete y-ion series and complementary b-ion series present.

### Protein classification and hierarchical cluster analysis

To determine the functions of identified proteins, we searched against the NCBI database (http://www.ncbi.nlm.nih.gov/) (accessed on 8 August 2012) and UniProt database (http://www.ebi.uniprot.org/). By integrative analysis of all the information collected from aforementioned processed, each protein was classified into certain functional category defined by us. Log (base 2) transformed ratios were used forhierarchical clustering analysis using Cluster 3.0 available onthe Internet (http://bonsai.hgc.jp/~mdehoon/software/cluster/software.htm), and the results were visualized using Java TreeView (http://jtreeview.sourceforge.net/).

### Protein subcellular location prediction

The subcellular location of the identified proteins was predicted using five internet tools: (1) YLoc (http://abi.inf.uni-tuebingen. de/Services/YLoc/webloc.cgi), confidence score ≥0.7; (2) LocTree3 (https://rostlab.org/services/loctree3/), expected accuracy ≥80%; (3) ngLOC (http://genome.unmc.edu/ngLOC/ index.html), probability ≥80%; (4) TargetP (http://www.cbs.dtu.dk/services/TargetP/), reliability class ≤3; (5) Plant-mPLoc (http://www.csbio.sjtu.edu.cn/bioinf/plant-multi/) no threshold value in Plant-mPLoc. Only the consistent predictions from at least two tools were accepted as a confident result. For the inconsistent prediction results among five tools, subcellular localizations for corresponding proteins were predicted based on literatures.

### Total RNA extraction, reverse transcription and qReal-Time (qRT)-PCR analysis

Based on the findings of proteomic analysis, we chose 12-responsive proteins which might be the underlying regulator of WD tolerance for qRT-PCR analysis for the verification of proteomic data. Total RNA from abdominal adipose tissue was isolated using Trizol reagent. One-microgram total RNA was performed in reverse transcription with Revert Aid Reverse Transcriptase (Fermentas) and Oligo d (T) primers (TaKaRa). Reverse transcription conditions for each cDNA amplification were 65 °C for 5 min, 37 °C for 52 min, and 70 °C for 15 min. Real-time RT-PCR was carried out using the Real-time PCR System (Roche Light Cycler 480 II, Switzerland) and SYBR Premix Ex Taq (TaKaRa). The primers used for the PCR are listed in Additional file 1: Table S1. Values represent the mean of three biological replicates and two technical replicates. The relative gene expression levels were calculated by the 2^-ΔΔt^ method [35].

### Data analysis

All results were presented as means ± standard error (SE) of at least three replicates. Results were analyzed by one-way ANOVA using the statistical software SPSS 17.0 (SPSS Inc. Chicago, IL, USA). Posthoc comparisons were tested using the LSD test at a significance level of *p* < 0.05.

## Results

### Effects of WD on photosynthesis

The photosynthesis indexes of *C. humilis* under WD were analyzed. After 24 days of treatment, Pn, Gs and Ci in *C. humilis* leaves under WD were significantly lower than those under control conditions (Table 1). In addition, chlorophyll fluorescence parameters were monitored to determine the performance of photosystem II (PSII) photochemistry. Fv/Fm were not significantly altered after 6 days WD, but were significantly reduced by 7 and 14% at 12 and 24 days WD, respectively, as compare with controls (Table 1).

**Table 1:** Effects of Pn, Gs, Ci and Fv/Fm of *C. humilis* under WD for 0, 6, 12, and 24 d

Treatment time (d)	Pn		Gs		Ci		Fv/Fm	
	(μmol CO_2_ · m^-2^ · s^-1^)		(μmol H_2_O_2_ · m^-2^ · s^-1^))		(μmol CO_2_ · m^-2^ · s^-1^)			
	Control	WD	Control	WD	Control	WD	Control	WD
0	22.3 ± 0.4a	21.6 ± 0.6a	187 ± 22a	192 ± 23a	352 ± 7a	338 ± 26a	0.838 ± 0.03a	0.829 ± 0.013a
6	21.9 ± 0.4a	18.6 ± 0.8b	199 ± 11a	177 ± 16a	345 ± 9a	321 ± 12ab	0.837 ± 0.05a	0.796 ± 0.018ab
12	22.6 ± 0.7a	15.3 ± 0.9c	189 ± 12a	116 ± 9c	342 ± 12a	311 ± 21bc	0.830 ± 0.06a	0.774 ± 0.017b
24	20.8 ± 1.1a	13.6 ± 0.1d	187 ± 33a	86 ± 11d	354 ± 14a	288 ± 17c	0.822 ± 0.06a	0.716 ± 0.022c

Each value is the mean ± SE of five independent experiments. The different small letters indicate significant difference (*p*<0.05) under WD

### Effects of WD on leaf relative water content (LRWC) and membrane lipid peroxidation

**Fig. 1:**
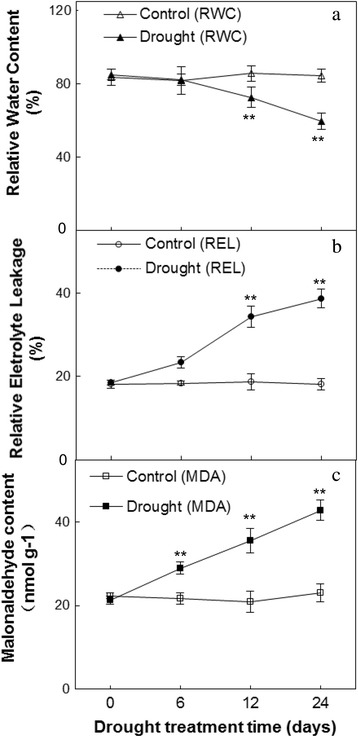
Effects of water deficit on leaf relative water content (LRWC) (a), relative electrolyte leakage (REL) (b), and malondialdehyde (MDA) (c) in *C. humili* leaves. Values are presented as means ± SE (*n* = 3). The values were determined under control and water deficit treatment at 0, 6, 12, and 24 days. * and ** indicate values that differ significantly from controls at *p* ≤ 0.05 and *p* ≤ 0.01, respectively, according to LSD test

WD resulted in a gradual decrease in LRWC in *C. humilis,* although there were no significantly changes of LRWC in WD treated plants in the early treatment period (6 d), as compared with controls. At 12 and 24 d of WD, LRWC was decreased to 85 and 71% of controls, respectively (Fig. 1a).

To determine the levels of membrane integrity and permeability of the cell membrane in *C. humilis* seedlings during WD, the status of REL and MDA were monitored. Leaf REL and MDA content increased slightly in the early WD period (6 d) (Fig. 1b, c), and *C. humilis* plants had a significantly high REL level by the end of WD treatment (24 d). These imply that the membrane integrity of WD plants was destroyed and the electrolyte inside of cells was come out to some extents.

### Effects of WD on soluble sugar and proline

**Fig. 2:**
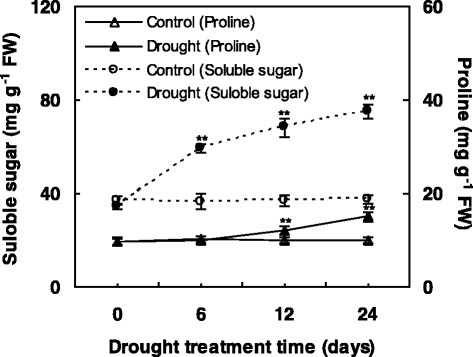
Effects of water deficit on leaf soluble sugar and proline contents in *C. humili*leaves. Values are presented as means ± SE. The values were determined under control and water deficit treatment at 0, 6, 12, and 24 days. * and ** indicate values that differ significantly from controls at *p* ≤ 0.05 and *p* ≤ 0.01, respectively, according to LSD test

Under WD, plants could synthesize compatible low-molecular weight organic solutes, such as proline and soluble sugar to alleviate WD-induced osmotic stress. In this study, the soluble sugar content increased significantly during the whole WD period compared to controls (Fig. 2). The proline content did not display significantly changes in the early treatment period, while they showed dramatic increases upon 12 and 24 d of WD (Fig. 2). The accumulation of soluble sugar and proline contents may contribute to maintain osmotic balance under WD.

### Effects of WD on H_2_O_2_ content, the generation rate of O_2_^-^ and antioxidant enzyme activities

**Fig. 3:**
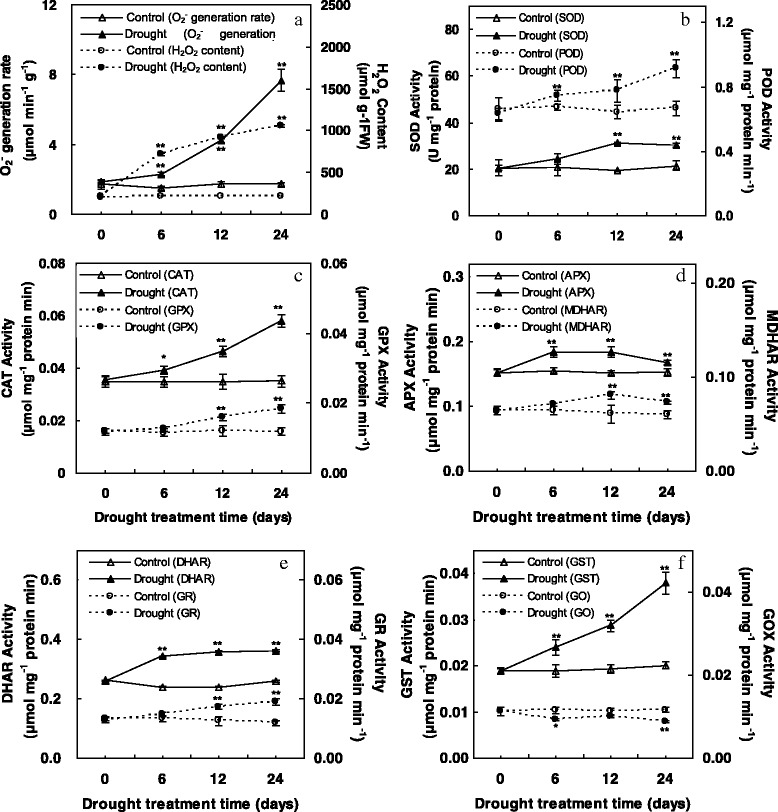
Effect of water deficit on the activities of antioxidant-related enzymes in *C. humili*leaves. **a** O_2_^-^ generation rate and H_2_O_2_ content; **b** superoxide dismutase (SOD) and peroxidase (POD); **c** catalase (CAT) and glutathione peroxidase (GPX); **d** ascorbate peroxidase (APX) and monodehydroascorbate reductase (MDHAR); **e** dehydroascorbate reductase (DHAR) and glutathione reductase (GR); **f** glutathione S-transferase (GST) and glycolate oxidase (GO). Values are means ± SE. based on four independent determinations after plants were treated for 0, 6, 12, and 24 days of water deficit, and *bars* indicate standard deviations. * and ** indicate values that differ significantly from controls at *p* ≤ 0.05 and *p* ≤ 0.01, respectively, according to LSD test

In the present study, the rate of O_2_^-^ generation and H_2_O_2_ contents were increased gradually on time for *C. humilis* leaves subjected to WD (Fig. 3a), suggesting that oxidative stress was occurred. The activity of SOD, a key enzyme of scavenging ROS, was enhanced in *C. humilis* leaves exposed to WD (Fig. 3b), which might enable the dismutation of superoxide into oxygen and H_2_O_2_ timely. Plants possess a complex mechanism of enzymatic antioxidants (e.g. POD, CAT, GPX, APX, MDHAR, DHAR, GR, and GST) that protect cells from oxidative damage by scavenging ROS. Thus, their activities were examined in this study. The activities of POD, CAT, APX, DHAR and GST were significantly increased in the whole WD period (Fig. 3b, c, d, e, f). The activities of GPX, MDHAR, and GR were increased after 12 d of WD conditions (Fig. 3c, d, e). In contrast, the GOX activities were decreased under WD (Fig. 3f), although the decrease extents were less significant as compared to other enzymes. In the whole level, the induction of enzymes activities under WD implies that they play a crucial role in defending against the oxidative stress and cell damage induced by WD.

### Identification, functional categorization and subcellular location of different changed proteins in response to WD

**Fig. 4:**
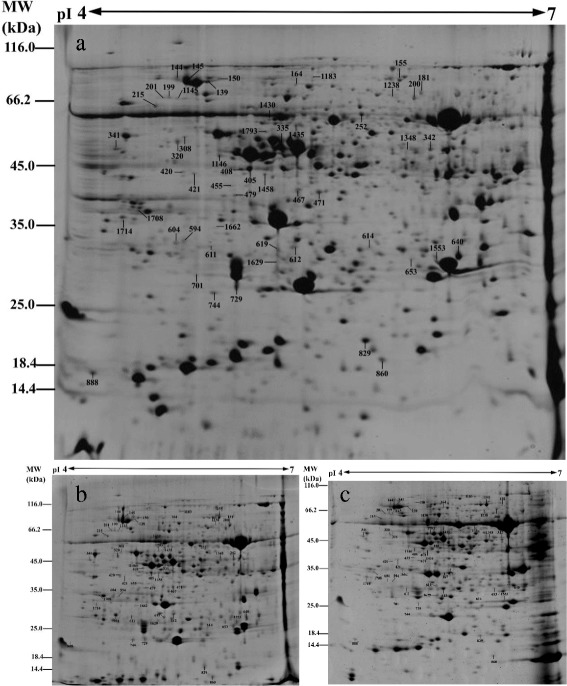
Representative 2-DE gel image of proteins in response to water deficit from *C. humili*leaves. **a** control; **b** 12 days after water deficit; **c** 24 days after water deficit. Proteins were separated on 13 cm IPG strips (pI 4-7 linear gradient) using IEF in the first dimension, followed by 12.5% SDS-PAGE gels in the second dimension. The 2-DE gel was stained with Coomassie Brilliant Blue. Molecular weight (MW) in kDa and pI of proteins are indicated on the left and top of the gel, respectively. A total of 46 differentially expressed proteins identified by MALDI-TOF-MS/MS were marked with numbers on the gel, and detailed information can be found in Additional file 2: Table S2 and Table 1

**Fig. 5:**
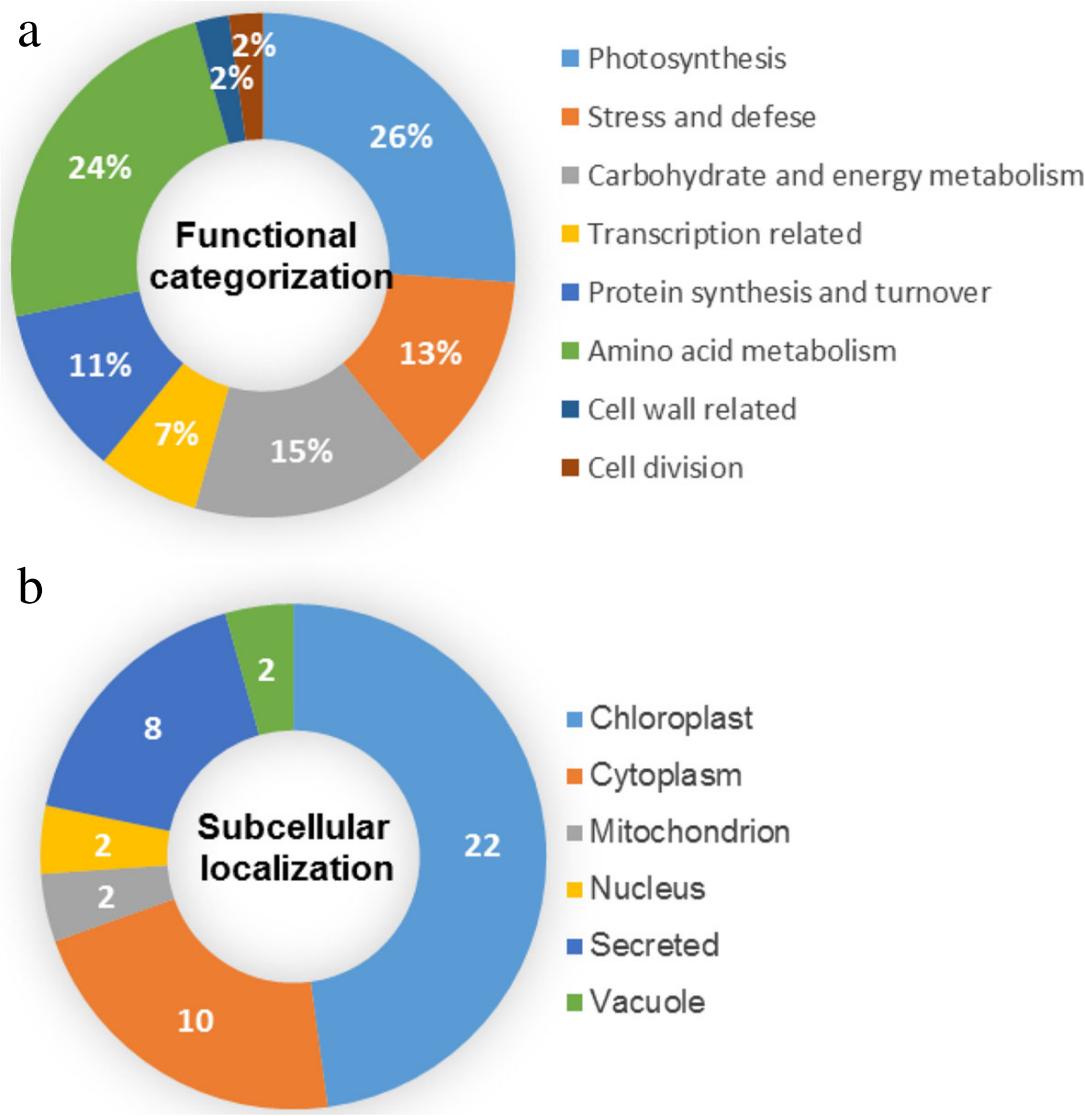
Functional categorization and subcellular localization of the differentially expressed proteinsat different water deficit time points of *C. humili*leaves. **a** A total of 46 DEPs were classified into eight functional categories. The percentage of proteins in different functional categories is shown in the pie. **b** Subcellular localization categories of the identified proteins. The numbers of proteins with different locations are shown

Based on the results of biochemical assays, we chose 0, 12, and 24 d WD samples for proteomic analysis. Total soluble proteins from control and WD-treated *C. humilis* leaves were separated and imaged, respectively (Fig. 4). Approximately, 700 Coomassie Brilliant Blue-stained protein spots were detected on the pI 4-7 gels. Only the protein spots that exhibited reproducible changes under WD (average fold changes >1.5 or <0.6, *p*-value < 0.05) were retained for further analysis. A total of 46 protein spots showed significant changes in WD samples as compared to control leaves (Table 2). The differentially expressed protein spots were successfully identified by using tandem MS, of which 46 spots represented 43 unique proteins (Table 2, Additional file 2: Table S2). Further examination of their electrophoretic patterns indicated that their inferred mass or isoelectric point values differed, perhaps owing to post-translational modification or degradation. To further examine the differentially expressed proteins, the identified proteins were assigned to Gene Ontology terms (Table 2, Additional file 2: Table S2). The 46 IDs were classified into 8 categories (Fig. 5a), covering a wide range of biological processes, which include photosynthesis (26%), stress and defense (13%), carbohydrate and energy metabolism (15%), transcription related proteins (7%), protein synthesis and turnover (11%), amino acid metabolism (24%), cell wall related (2%), and cell division (2%), respectively. In total, 22 significantly changed proteins were predicted to be localized in chloroplast, 10 in cytoplasm, two in mitochondria, two in nucleus, eight in secreted, and two in vacuole (Fig. 5b, Additional file 3: Table S3).

**Table 2:**
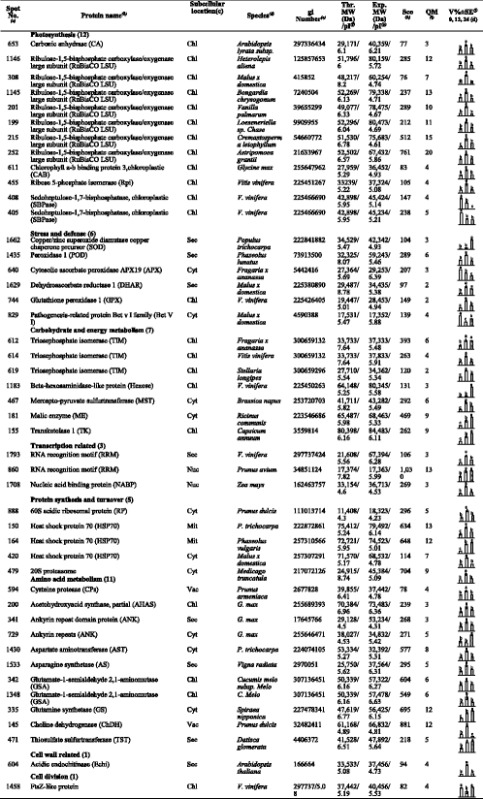
Relative protein content changes in *C. humilis* leaves under water deficit

^*a*^Assigned spot number as indicated in Fig. 4. ^*b*^The name and functional categories of the proteins using MALDI TOF-TOF MS. ^*c*^Protein subcellular localization predicted by softwares (YLoc, LocTree3, Plant-mPLoc, ngLOC, and TargetP). Only the consistent predictions from at least two tools were accepted as a confident result listed in Additional file 3: Table S3. Chl, chloroplast; Cyt, cytoplasm; Mit, mitochondria; Nuc, nucleus; sec, secreted; vac, vacuole.^*d*^The plant species that the peptides matched. ^*e*^Database accession numbers from NCBInr. ^*f, g*^Theoretical (e) and experimental (f) mass (kDa) and pI of identified proteins. Experimental values were calculated using Image Master 2D Platinum Software. Theoretical values were retrieved from the protein database. ^*h*^The Mascot score obtained after searching against the NCBInr database. ^*i*^The number of unique peptides identified for each protein. ^*j*^ The mean values of protein spot volumes relative to total volume of all the spots. Four water deficit (0 d, 12 d, and 24 d) were performed. Error bars indicate mean ± standard error (SE)

### Hierarchical clustering of different changed proteins in response to WD

**Fig. 6:**
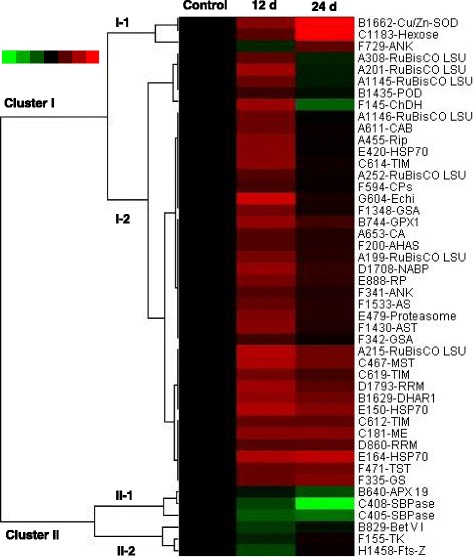
Dendrogram of 46 differentially abundant proteins obtained by hierarchical clustering analysis. The three columns represent different drought treatment time points, including 0, 12, and 24 days. The rows represent individual proteins. Two main clusters (I and II) and subclusters of I and II (I-1, I-2, II-1, and II-2) are shown on the left side. Functional categories indicated by capital letters, spot numbers, and protein name abbreviations are listed on the right side. The scale bar indicates log (base2) transformed protein abundance ratios ranging from -3.0 to 3.0. The ratio was calculated as protein abundance at control divided by abundance each treatment, respectively. The increased and decreased proteins are represented in red and green, respectively. The color intensity increases with increasing abundant differences. Undetected proteins are indicated in gray. Abbreviations for functional categories: A, Photosynthesis; B, Stress and defense; C, Carbohydrate and energy metabolism; D, Transcription related; F, Protein synthesis and turnover; E, Amino acid metabolism; I, Cell wall related; J, Cell division. Detailed information on protein names and abbreviations can be found in Table 1

To study protein expression characteristics in each functional category, hierarchical clustering analysis was performed that yielded two main clusters (Fig. 6). Cluster I included 40 IDs (Fig. 6), the levels of which were increased under WD. These proteins were divided into two subclusters: Subcluster I-1 contained the proteins mainly increased significantly under 24 d WD, while subcluster I-2 contained the proteins mainly increased under 12 d WD. The proteins in cluster I covered seven function categories. The remaining six IDs were grouped into cluster II, representing decreased proteins under WD. Cluster II contained two subclusters: Subcluster II-1 contained 3 IDs induced either at 12 and 24 d of WD, while subcluster II-2 included 3 proteins significantly increased only at 12 d of WD (Fig. 6). When examined proteins in each cluster, most of increased IDs were involved in photosynthesis and ROS scavenging and were mainly included in cluster I, whereas carbohydrate and cell division were decreased and present in cluster II.

### Homologous gene expression of different changed proteins

**Fig. 7:**
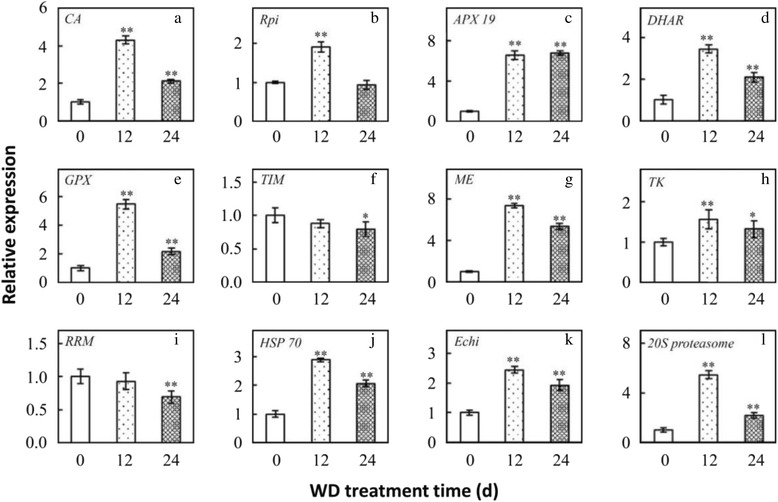
qRT-PCR analysis of gene expression of WD response proteins in *C. humilis*. RNA was extracted from WD treatment for 0, 12, and 24 h days, respectively. Values represent the mean of three biological replicates and two technical replicates. Each data point represents mean ± SE (*n* = 3). * and ** indicate values that differ significantly from controls at *p* ≤ 0.05 and *p* ≤ 0.01, respectively, according to LSD test. CA, carbonic anhydrase; Rpi, Ribose 5-phosphate isomerase; DHAR, dehydroascorbate reductase; GPX, glutathione peroxidase; TIM, triosephosphate isomerase; ME, malic enzyme; TK, transketolase; RRM, RNA recognition motif; HSP70, heat-shock protein 70; Echi, acidic endochitinase

To determine whether changes of gene transcription levels correlated with changes of protein levels, a qRT-PCR analysis of 12 genes was performed (Fig. 7). The results demonstrated that nine genes, namely carbonic anhydrase (CA), Ribose 5-phosphate isomerase (Rpi), dehydroascorbate reductase (DHAR), glutathione peroxidase (GPX), malic enzyme (ME), transketolase 1 (TK), heat shock protein 70 (HSP 70), 20S proteasome, and acidic endochitinase (Echi) showed consistent expressional trends with their homologous proteins (Fig. 7, Table 2). They were involved in photosynthesis, stress and defense, carbohydrate and energy metabolism, protein synthesis and turnover, and cell wall related, respectively. In addition, three genes, namely cytosolic ascorbate peroxidase (APX19), triosephosphate isomerase (TIM), and RNA recognition motif (RRM) appeared opposite expressional trends with homologous proteins (Fig. 7, Table 2). They were involved in stress and defense, carbohydrate and energy metabolism, and transcription, respectively. The results of correlation analysis indicate that the aforementioned metabolic processes were modulated by post-transcriptional and/or post-translational regulation during WD. The inconsistent abundances of transcripts and proteins in WD also support the notion that pre-synthesized mRNA and proteins would function in the process of WD.

## Discussion

WD is the main limiting factor for plants growing in arid areas. Biochemical, physiological and molecular influences on plants are wide-spread during WD and can be divided into three aspects: growth control, stress damage control and osmotic homeostasis [2, 36]. An integrated proteomics, biochemical, physiological and morphological approach was used for our research to investigate these aspects of WD responses in *C. humilis*.

### Photosynthetic acclimation to WD

**Fig. 8:**
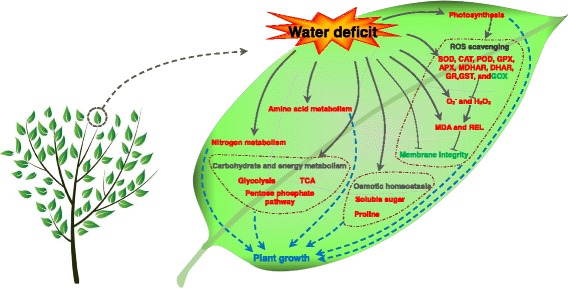
Schematic presentation of systematic in *C. humili*leaves under water deficit. Water deficit inactive photosynthesis and lead to ROS burst, which resulting in the damage to cell membrane. To alleviate ROS toxicity, specific ROS scavenging pathways (e.g., APX, MDHAR, DHAR, GR, GST, GPX, POD, and CAT pathways) are induced. Water stress induces glycolysis, TCA cycle, and pentose phosphatepathway, providing carbon and energy in stressed leaves. In addition, the accumulation of various osmolytes, such as proline and soluble sugar are enhanced. Importantly, water deficit increases the nitrogen and amino acid metabolisms. Solid line with arrow and “T” shape line represent stimulation and inhibition, respectively. The red and green words indicate water deficit-induced and water deficit-reduced cellular processes, respectively. Dashed lines indicate indirect regulations. Abbreviations: APX, ascorbate peroxide; CAT, catalase; DHAR, dehydroascorbate reductase; H_2_O_2_, hydrogen peroxide; MDA, malondialdehyde; MDHAR, monodehydroascorbate reductase; GOX, glycoxylate oxidatase; GPX, glutathione peroxidase; GR, glutathione reductase; GST, glutathione-s-transferases; O_2_^-^, superoxide anion; POD, peroxidase; REL, relative electrolyte leakage; ROS, reactive oxygen species; SOD, superoxide dismutase

In *C. humilis* seedlings, Gs and Ci were declined gradually under WD, indicating that the decrease in Pn (Table 1) may be the result of a stomatal limitation [37]. Based on our determination of chlorophyll fluorescence, Fv/Fm significantly declined in response to WD at 12 and 24 days (Table 1), suggesting that WD causes photoinhibition of PSII. To our surprise, we found that 10 out of 12 light reaction and Calvin cycle-related proteins, including a carbonic anhydrase (CA), seven ribulose-1,5-bisphosphate carboxylase/oxygenase large subunits (RuBisCO-LSU), a Ribose 5-phosphate isomerase (Rpi) and a chlorophyll a-b binding protein (CAB) were accumulated under WD (Table 2, Fig. 8). Among them, Chlorophyll a-b binding protein (CAB), PSI light harvesting chlorophyll binding protein, is the intrinsic transmembrane antenna proteins (Lhca’s) occurring in the reaction center of PSI. PSI is known to be the most efficient light converter in nature since pigments in the PSI are not being quenched and energy transfer to the electron donor is very rapid [38]. Plastocyanin functions as an electron transfer agent between cytochrome f and P700^+^ from PSI [39]. We speculated that the increased CAB could transfer more excitation energy to the reaction center, and the accumulation of plastocyanin can donate more electrons to PSI. Finally, CAB with a higher abundance may help to minimize the energy loss caused by a reduction of PSII efficiency in the *C. humilis* response to moderate WD. In addition, CAs and RuBisCO were increased under 12 and 24 d WD in our study (Table 2). This indicated that WD promoted the CO_2_ assimilation, leading to the reprogramming of carbon metabolism. Carbonic anhydrase (CA), at the donor side of PSII, can aid to increase the concentration of CO_2_ within the chloroplast, which induces the carboxylation rate of RuBisCO [40]. RuBisCO is an important enzyme involved in the first major step of carbon fixation [41, 42]. It has been reported that overexpression of the CA in *Arabidopsis* resulted in an increase in plant biomass [41]. Previous proteomics studies have also revealed that the increase of Calvin cycle-related and light reaction proteins in *Cynodon dactylon* [18] and *Malus* [28].

### Regulation of osmostasis and redox homeostasis to cope with WD

In our results, the accumulation of osmolytes in *C. humilis* enhanced the positive response to WD (Fig. 1b, c; Fig. 2). The regulation of osmostasis and redox homeostasis are critical for WD tolerance [43]. And WD affects cell membrane integrity and membrane lipid composition, resulting in the changes of REL and MDA content [36, 44]. This often happened in other species, such as *Amygdalus mira (Koehne) Yü et Lu* [44] and rice [45]. To cope with osmotic imbalance and protect membrane, diverse compatible osmolytes were accumulated in cells. On the other hand, proline is involved in radical scavenging, which has an important defensive role on resisting the WD-induced oxidative stress [46]. Total soluble sugar also acts as a crucial array of prevention and signals that is helpful to sense and control photosynthetic activity and ROS balance [47].

To minimize the damaging effects of ROS, plants have evolved various antioxidant enzymes defense pathways [48–50]. In this study, we found that the activities of several enzymes such as SOD, POD, CAT, GPX/GST, and four key enzymes of ascorbate–glutathione (AsA-GSH) cycle, were affected by WD in *C. humilis* (Fig. 3). All these implied that AsA-GSH cycle and GPX/GST pathway were enhanced in *C. humilis* for WD tolerance [51]. Interestingly, the activity, expression, and abundance of some enzymes were inconsistent. Our proteomics results showed that the abundances of SOD, POD, DHAR, and GPX were increased, except that the abundances of APX were decreased. However, qRT-PCR results found that the expression of APX, DHAR, and GPX were increased (Fig. 7). Enzyme abundances may be inconsistent with their activities and expressions, as the activity is also modulated by the protein conformation and post-translational modifications. This indicated that ROS scavenging enzymes in *C. humilis* seedlings were modulated at both translational and post-translational levels for WD tolerance.

### WD-responsive transcription, protein synthesis, and protein processing

In response to WD, plants exhibit quick switches from metabolic quiescent state to active state. In this study, our proteomics results revealed that two RNA recognition motif (RRM) and a nucleic acid binding protein (NABP) were increased at 12 and 24 days treatment. Besides, a ribosomal protein was increased at 12 d WD (Table 2). These indicated that RNA processing and protein synthesis-regulated metabolic increase would be a positive response to WD. During gene expression, RNA processing by RNA chaperone is critical for keeping the proper RNA structure and function in response to WD [52].

We found three HSP 70s were WD-responsive in *C. humilis* at 12 and 24 d WD (Table 2). Protein folding and processing were active for preventing WD-induced denature and incomplete aggregation [45], and HSP 70 belongs to the conservative family of molecular chaperones, and exists in all cells and organs, assisting protein folding, aggregation, translocation, and degradation [53]. Thus, the abundance of HSP 70 was significantly elevated in order to eliminate misfolded proteins. It has been also also revealed that 26S proteasome increased in response to drought stress (Table 2), which is important to remove abnormal or damaged proteins and to control the levels of certain regulatory proteins during drought stress. Similar study also found in *Hordeum vulgare* [54] and *Medicago sativa* [55], respectively. These findings indicate that the enhancement of the ubiquitin/26S proteasome system is important for plants to cope with drought.

### Enhancement of carbohydrate supply and other specialized metabolism under WD

In this study, five IDs were affected by WD, including three triosephosphate isomerases (TIM) involved in glycolysis, one transketolase (TK) involved in pentose phosphate pathways (PPP), and malic enzymes (ME) involved in TCA cycle (Table 2 and Fig. 8). The regulation of carbohydrate metabolism is an important strategy for plants to respond to WD [56]. TIM catalyzes the interconversion of glyceraldehyde-3-phosphate to dihydroxyacetone phosphate [57]. TK, key enzymes of the reductive and oxidative pentose phosphate pathways, are responsible for the synthesis of sugar phosphate intermediates [58]. Malic enzymes (MEs) involved in malate dehydrogenase (MDH) system catalyze the L-malate decarboxylation reaction through their oxidation, and MDH catalyzethe interconversion of malate and oxaloacetate in areversible reaction of the TCA cycle have an important role in biochemical adaptation of plants to stress [59]. In addition, TK is considered to participate in pentosephosphate pathway. The pentose phosphate pathway is important to maintain carbon homoeostasis, to provide precursors for nucleotide and amino acid biosynthesis, and to provide reducing molecules for defeating oxidative stress Stincone et al. [60]. TK is the key enzyme of the non-oxidative branch of the pentose phosphate pathway of carbohydrate transformation [61]. Accumulation of TK would promote the enhancement of the non-oxidative branch, yielding ribose 5-phosphate for the synthesis of nucleic acids and amino acids accompanied by the production of NADPH, which is critical to maintain redox balance under stress situations [62]. Besides, our results are consistent with previous proteomic studies in *Phaseolus vulgaris* [63], rice [64], and wheat [65] under WD. Previously, enhancement of the glycolysis, PPP, and TCA has been found in several species in response to various stress conditions [66], which would provide more glyceraldehydes-3-phosphate (G3P), glucose-6-phosphate (G6P), NADPH, and erythrose-4-phosphate (E4P) that could be used to produce more ATP for maintaining the basic metabolism under stress [66, 67]. This might be a strategy for plant to enhance the ability of seedlings to survive under WD, which makes it possible for the cell to adapt to its metabolic needs.

### Rapid nitrogen and amino acid metabolisms are essential for WD

Nitrogen assimilation is affected by abiotic stress in plants. In this process, exogenous absorbed nitrate is transformed to ammonium by nitrate reductase (NR) and nitrite reductase (NIR), and then assimilated by glutamine synthetase (GS) and glutamate synthase (GOGAT) into amino acids [18, 68]. Our proteomics results revealed that 11 IDs were increased under WD (Table 2 and Fig. 8). Among them, the up-regulated 3-mercaptopyruvate sulfurtransferase (MST) implied that the efficiency of cellular redox state was increased. Related research showed that it catalyzes pyruvate transsulfuration from 3-mercaptopyruvate, which transamined from cysteine and also contributes to maintain the cellular redox state [69]. Similarly, the increased glutamine synthetase could be involved in the osmotic stress response, as glutamine synthetase is related to proline biosynthesis [70]. In addition, the accumulation of asparagine synthetase (AS) and aspartate aminotransferase (AST) in the present study might aid to nitrogen and amino acid metabolisms, leading to resist to WD response. AS and AST are key enzymes involved in carbon and nitrogen distribution [71]. AS is necessary for the production of nitrogen-rich amino acid asparagine, which is the primary of nitrogen metabolism [72–74]. Similarly, the previous study found that influence of over-expression of cytosolic AST on amino acid metabolism and defence responses against *Botrytis cinerea* infection in *Arabidopsis thaliana* [75].

## Conclusion

This study is the first proteomic analysis in *C. humilis* response to WD to our knowledge. In general, we found some responsive pathways are pivotal under WD as shown in Fig. 8 by integrative analysis of all the results from physiological and proteomic analysis in *C. humilis*. Firstly, photosynthesis is strongly affected by WD. The increase of Calvin cycle-related and light reaction proteins may help to convert more light energy and minimize the energy loss caused by a reduction of PSII efficiency. In addition, the reestablishment of osmostasis and redox homeostasis, reprogramming of nuclear and chloroplast gene expression and protein processing are positive for *C. humilis* to WD. Furthermore, nitrogen and amino acid metabolisms lead to resist to WD response. These findings provide important information for understanding WD-responsive mechanisms in *C. humilis* seedlings.

## Additional files

Additional file 1: Table S1. Primers used for the quantitative real-time RT-PCR analysis. (XLSX 9 kb)

Additional file 2: Table S2. Differentially expressed proteins and the sequences of peptides identified in *C. humilis* under WD using MALDI TOF-TOF MS. (XLSX 48 kb)

Additional file 3: Table S3. The subcellular localization prediction of the differentially expressed proteins identified in *C. humilis* under WD. (XLSX 30 kb)

## Abbreviations

2-DE: Two dimensional electrophoresis
ACN: Acetonitrile
DTT: Dithiothreitol
GAPDH: Glyceraldehyde 3-phosphatedehydrogenase
MALDI-TOF/TOF: Matrix-assisted laser desorption/ionization time-of-flight/time-offlight
RuBisCO: Ribulose bisphosphate carboxylase/oxygenase
TCA: Tricarboxylic acid
TFA: Trifluoroacetic acid
